# GLP-1 analogues for neuroprotection after out-of-hospital cardiac arrest: study protocol for a randomized controlled trial

**DOI:** 10.1186/s13063-016-1421-2

**Published:** 2016-06-30

**Authors:** Sebastian Wiberg, Christian Hassager, Jakob Hartvig Thomsen, Martin Frydland, Dan Eik Høfsten, Thomas Engstrøm, Lars Køber, Henrik Schmidt, Jacob Eifer Møller, Jesper Kjaergaard

**Affiliations:** Department of Cardiology, Copenhagen University Hospital, Rigshospitalet, Copenhagen, Denmark; Department of Anaesthesiology and Intensive care, Odense University Hospital, 5000 Odense C, Denmark; Department of Cardiology, Odense University Hospital, 5000 Odense C, Denmark

**Keywords:** Out-of-hospital cardiac arrest, Neuroprotection, GLP-1 analogues

## Abstract

**Background:**

Attenuating the neurological damage occurring after out-of-hospital cardiac arrest is an ongoing research effort. This dual-centre study investigates the neuroprotective effects of the glucagon-like-peptide-1 analogue Exenatide administered within 4 hours from the return of spontaneous circulation to comatose patients resuscitated from out-of-hospital cardiac arrest.

**Methods/design:**

This pilot study will randomize a total of 120 unconscious patients with sustained return of spontaneous circulation after out-of-hospital cardiac arrest undergoing targeted temperature management in a blinded one-to-one fashion to a 6-hour and 15-minute infusion of either Exenatide or placebo. Patients are eligible for inclusion if resuscitated from cardiac arrest with randomization from 20 minutes to 240 minutes after return of spontaneous circulation. The co-primary endpoint is feasibility, defined as the initiation of treatment within the inclusion window in more than 90 % of participants, and efficacy, defined as the area under the neuron-specific enolase curve from 0 to 72 hours after admission. Secondary endpoints include all-cause mortality at 30 days and Cerebral Performance Category as well as a modified Rankin Score at 180 days. The study has been approved by the Danish National Board of Health and the local Ethics Committee and is monitored by Good Clinical Practice units. The study is currently enrolling.

**Discussion:**

This paper presents the methods and planned statistical analyses used in the GLP-1 trial and aims to minimize bias and data-driven reporting of results.

**Trial registration:**

1) Danish National Board of Health, EudraCT 2013-004311-45. Registered on 25 March 2014.

2) Videnskabsetisk komité C, Region Hovedstaden, No. 45728. Registered on 29 January 2014.

3) Clinicaltrial.gov, NCT02442791. Registered on 25 of January 2015.

**Electronic supplementary material:**

The online version of this article (doi:10.1186/s13063-016-1421-2) contains supplementary material, which is available to authorized users.

## Background

While the mortality after out-of-hospital cardiac arrest (OHCA) has improved over the last decade, mortality remains as high as 90 % [1], and even after successful resuscitation and admission to an intensive care unit (ICU), the in-hospital mortality is 30–50 % [2, 3]. Anoxic brain injury remains the leading cause of death in these patients [4]. The mechanisms causing neurological damage are complex but involve both ischemia and reperfusion injury leading to tissue degeneration and loss of neurological function, the extent of which depends on duration and density of the insult [5]. Targeted temperature management (TTM) may attenuate this damage in an experimental setting [6–9], and clinical trials have shown promising results in improving neurological function and survival [10, 11]. Despite this, the optimal target temperature is debated [12], and active neuroprotection in addition to temperature management seems intuitively beneficial. Glucagon-like peptide-1 (GLP-1) analogues are approved for the treatment of type 2 diabetes but have recently been suggested to ameliorate degenerative neurological disease and reduce inflammation after ischemic cerebral stroke [13]. The GLP-1 analogue Exenatide has been shown to reduce infarct volume after focal brain ischemia in mice [14] and to reduce infarct size in a model of acute myocardial infarction (MI) and reperfusion in swine [15]. In humans, patients treated with Exenatide after MI have been shown to have a larger salvage index assessed by cardiac magnetic resonance imaging (MRI) after 3 months [16, 17]. These findings have been confirmed by later trials [18, 19]. In addition, Exenatide has been well tolerated in acutely ill patients with ST-elevation myocardial infarction with no apparent increased risk of adverse events, including hypoglycaemia or pancreatitis, compared to placebo [17].

The present trial will investigate the neuroprotective capabilities of the GLP-1 analogue Exenatide administered for 6 hours and 15 minutes within 4 hours after the return of spontaneous circulation (ROSC) in comatose patients resuscitated from OHCA.

## Methods/design

This pilot study is a double-blind, randomized, placebo-controlled, clinical trial assessing the effect of Exenatide compared with placebo on top of TTM in adult comatose OHCA patients. Patients are enrolled at two tertiary Danish university hospitals offering highly specialized cardiac care for a population of approximately 3,000,000 citizens.

### Trial procedure

PHASE 1 (hospital admission to start of intervention): Unconscious patients who have been admitted to the hospital with sustained ROSC after OHCA are eligible for screening. The inclusion window is 220 minutes, i.e. from 20 minutes after ROSC (defined as ‘sustained ROSC’) until 240 minutes after ROSC. The patient’s eligibility for trial inclusion is assessed according to inclusion and exclusion criteria (Table 1). If all inclusion criteria and no exclusion criteria are met, informed consent is obtained, in accordance with Danish legislation, from two independent medical doctors not involved with the trial. Patients are then randomly allocated to active study drug or placebo via an internet-based randomization algorithm available on the trial website. Baseline characteristics are obtained.

**Table 1:** Inclusion and exclusion criteria

Inclusion criteria
1. Age ≥ 18 years
2. OHCA of presumed cardiac cause
3. Sustained ROSC, defined as ROSC when chest compressions have not been required for 20 consecutive minutes and signs of circulation persist
4. Unconsciousness (Glasgow coma scale (GCS) < 8) after sustained ROSC
Exclusion criteria
1. Conscious patient (GCS ≥ 8)
2. Female of childbearing potential, unless a negative human chorionic gonadotropin (HCG) test can rule out pregnancy within the inclusion window
3. In-hospital cardiac arrest (IHCA)
4. OHCA of presumed non-cardiac cause, e.g. after trauma, dissection/rupture of major artery or arrest caused by hypoxia (i.e. drowning or hanging)
5. Known bleeding diathesis (medically induced coagulopathy does not exclude patient)
6. Suspected or confirmed acute intracranial bleeding
7. Suspected or confirmed acute ischemic stroke
8. Unwitnessed asystole
9. Known limitations in therapy and do-not-resuscitate order
10. Known disease making 180-day survival unlikely
11. Known pre-arrest cerebral performance category score (CPC) of 3 or 4
12. > 4 hours (240 minutes) from ROSC to randomization
13. Systolic blood pressure < 80 mmHg in spite of fluid loading/vasopressor and/or inotropic medication and/or mechanical circulatory support^a^
14. Temperature on admission < 30 °C
15. Known allergy to GLP-1 analogues, including Exenatide
16. Known pancreatitis
17. Known ketoacidosis
18. Uncorrected blood glucose at admission < 2.5 mmol/l

^a^If systolic blood pressure is recovering during the inclusion window, the patient can be included

PHASE 2 (duration of intervention): Phase 2 starts when the study drug/placebo administration is initiated, most often coinciding with the initiation of TTM. Patients are sedated and mechanically ventilated. Patients are treated for 24 hours with an automated-feedback device with temperature control to achieve a target core temperature (bladder) of 36 °C. After 24 hours of TTM, patients are rewarmed to a core temperature of 37 °C with no more than 0.5 °C per hour. Phase 2 ends when sedation is withheld.

PHASE 3 (from end of intervention period to 72 hours after end of intervention period): Sedation is stopped or tapered after rewarming when temperature is at least at 37 °C. Normothermia of 37 °C +/−0.5 °C is maintained until 72 hours from cardiac arrest if the patient is still managed in the ICU and is comatose or sedated. However, weaning from ventilation will be attempted at the earliest possible time during this phase based on standard procedures for the discontinuation of mechanical ventilation. Blood is drawn at 24, 48 and 72 hours and is later analysed for the biomarkers neuron-specific enolase (NSE) and S100B – markers of cerebral injury. Neurological evaluation of patients who remain in a coma is performed by blinded physicians at 72 hours or later after the end of the intervention period.

PHASE 4 (72 hours after the end of the intervention period to 28 days after OHCA): Neurological status according to the Cerebral Performance Category scale (CPC-scale) [20], and vital status are evaluated daily in the ICU on days 1, 2, 3, 4, 5, 6 and 7 and/or at hospital discharge, whichever comes first.

PHASE 5 (hospital discharge/day 28 to end of trial): Vital status and neurological status are evaluated on days 30 and 90 and then by telephone interview on day 180. The evaluation is performed by a research nurse blinded to the intervention allocation. Vital status will be assessed at the end of the trial using the Danish civil registration system. See attached Additional file 2.

### Inclusion

Patients with sustained ROSC after OHCA are eligible for inclusion if fulfilling the criteria displayed in Table 1.

### Study drug administration

Preparation of study drug (Exenatide/placebo): Trained hospital nurses not taking part in the management of the patient will prepare infusion bags with Exenatide or placebo. At first notice of a potential candidate for inclusion in the trial, a set of study drug and placebo will be prepared. The infusions will be prepared as follows:

First, 1.5 ml is removed from 250 ml of isotonic NaCl and 1.5 ml of 20 % human albumin is added to prevent binding of the study drug to the infusion material. For every potentially eligible patient, two infusion kits will be prepared. One is a placebo kit as described, and one has 25 μg Byetta (Lilly, Exenatide) added. The two infusion kits are labelled ‘A’ or ‘B’ by random allocation in a computer-based randomization system and brought directly to the ICU. The random allocation will occur via the trial website with a specific logon for personnel involved in study drug preparation. The allocation ‘A’ and ‘B’ will be chosen at random by the trial website algorithm (randomization generated into dynamic blocks), stratified for site and will be unblinded by the end of trial.

The cardiologist responsible for the patient’s treatment screens the patient’s eligibility and obtains informed consent from legal representatives. If the patient is eligible for inclusion and informed consent is obtained from two independent physicians, the patient is randomized via the trial website to receive either infusion kit ‘A’ or ‘B’, and the other kit is discarded. The cardiologist orders the infusion to be given. The infusion is given in a central or peripheral intravenous line. The nurses preparing the infusion kits remain blinded to participant allocation, and the nurses initiating the study drug infusion (ICU nursing staff) remain blinded to the preparation of the infusion kits.

The study drug infusion is initiated by the ICU nursing staff as soon as possible at a rate of 72 ml/hour (0.12 μg Exenatide/minute) for 15 minutes (volume to be infused is 18 ml), followed by 26 ml/hour (0.043 μg Exenatide/minute) for an additional 6 hours (volume to be infused: 156 ml). This concludes the pharmacological intervention and thus a total of 17.4 μg of Exenatide is administered. As the allocation is blinded, the same infusion rates are used in the placebo arm. For safety reasons, blood glucose is monitored closely during the administration period and for the following 2 hours. Blood glucose and corrective glucose administration is reported. Additional management of the patients is at the discretion of the attending physicians. Thus, all trial participants, care providers, investigators and outcome assessors remain blinded to the intervention until completion of the trial. If a suspected unexpected serious adverse reaction (SUSAR) is found, the sponsor has the ability to unblind the patient’s intervention allocation through the physician responsible for the trial website, who is otherwise detached from the trial.

#### Monitoring of compliance

Infusion of study drug ‘A’ or ‘B’ will be recorded along with time of initiation of the study drug infusion. In addition any dose reduction or interruption of administration will be recorded. Reasons for not infusing the study drug per protocol and any dose reduction outside of ± 5 minutes from the scheduled time point are registered. Suspected adverse events, as well as the actions taken to correct these and their consequences, are recorded.

### Concurrent medication/treatment

Computed tomography of the brain/neck/head will only be performed as clinically indicated, not as a routine screening after admission. Patients will be treated with standard therapies for cardiac diseases. Coronary angiography, percutaneous interventions and/or open-heart surgery will be performed according to current guidelines at the discretion of the treating physician. Necessary cardiac interventions will not be delayed by the trial intervention; however, efforts will be made to maintain the study drug infusion and TTM during treatment.

### Logistics

The study is enrolling at two tertiary centres with experience in conducting clinical trials. All investigators have been trained and ‘good clinical practice’ (GCP) certified. Other involved personnel (i.e. ICU nurses) have been trained in their specific roles.

### Endpoints

The co-primary endpoints are as follows:

1. Feasibility, defined as > 90 % initiation of study drug administration within 4 hours following ROSC in patients eligible for inclusion
2. Efficacy, defined as the area under the NSE curve from admission to 72 hours post-admission. Missing data will be imputed.

Secondary endpoints are as follows:

1. Area under the S100B curve with daily measurements until 72 hours, and absolute NSE and S100B values at 48 hours. Missing data will be imputed.
2. Composite outcome of all-cause mortality and poor neurological function, defined as the modified Rankin Scale (mRS) 4–6 at 30 days (telephone assessment)
3. Vital status at 7 days and at least 180 days (end of the study) after OHCA by registry-based follow-up
4. Assessment of CPC and mRS at 90 days
5. Area under the creatine kinase MB (CK-MB) and troponin T curve from 0 to 24 hours. Missing data will be imputed.
6. Safety, defined as the cumulated incidence of serious adverse events (SAE) related to the study drug: death, need for mechanical hemodynamic support, hypoglycaemia < 3.0 mmol/l, pancreatitis (S-amylase > 3 UNL), need for renal replacement therapy in the first 3 days. In addition, hypoglycaemia and pancreatitis will be reported separately as additional safety.

Tertiary endpoints are as follows:

1. Left ventricular ejection fraction on last in-hospital echocardiography, stratified by the presence of acute myocardial infarction as the cause of cardiac arrest. Patients dying during the index administration in whom a post-TTM echocardiography is not available will be given the lowest LVEF score seen in each allocation group. Missing data will be imputed.
2. Presence of EEG findings associated with poor prognosis and EEG performed as part of per-protocol prognostication
3. Vital status at 180 days and telephone-based CPC and mRS at 180 days
4. Vital status at 180 days stratified for cause of death (i.e. neurological versus cardiovascular and other – will be adjudicated by two intensive-care consultants blinded to treatment allocation)

Blinded efficacy variables will be recorded at 3, 30, 90 and 180 days. The telephone-based assessment will be performed by a single trained study nurse, not otherwise involved in the care of patients. Other variables will be assessed and recorded in the case report forms (CRF) by a trained physician not involved in the care of patients. The NSE and S100B plasma levels will be measured in the hospital laboratory for each site and recorded in the CRF. The study drug allocation will remain blinded until all follow-up data have been recorded. All CRFs will be entered into the trial database. The quality of data entry will be evaluated by random samples as well as range checks for data values. Individual patient data will be handled as ordinary chart records and will be kept according to national legislation.

#### Data monitoring

A Data Safety Monitoring Committee (DSMC) will be assembled. The DSMC will be responsible for safeguarding the interests of trial participants, assessing the safety and efficacy of the interventions during the trial, and for monitoring the overall conduct of the clinical trial. The DSMC will provide recommendations about stopping or continuing the trial to the Steering Group (SG) of the trial. To contribute to enhancing the integrity of the trial, the DSMC may also formulate recommendations relating to the selection/recruitment/retention of participants, their management, improving adherence to protocol-specified regimens and retention of participants, and the procedures for data management and quality control.

The DSMC will be advisory to the SG. The SG will be responsible for promptly reviewing the DSMC recommendations, for deciding whether to continue or terminate the trial, and for determining whether amendments to the protocol or changes in trial conduct are required.

A statistician selected by the members of the DSMC will perform the interim analysis. The sponsor has the responsibility to report the overall number of SAEs and SUSARs monthly to the DSMC. The recommendations of the DSMC regarding stopping, continuing or changing the design of the trial should be communicated without delay to the SG of the trial.

DSMC membership has been restricted to individuals free of conflicts of interest. The source of these conflicts may be financial, scientific or regulatory in nature. Any DSMC members who develop significant conflicts of interest during the course of the trial should resign from the DSMC. One ‘Formal Interim Analysis’ meeting is planned to review data relating to the treatment efficacy, patient safety and quality of trial conduct.

The independent GCP units include data quality monitoring, informed written consent forms and adjudication of endpoints as well as results of NSE and S100B analysis. The protocol is adapted to the ‘Standard Protocol Items: Recommendations for Interventional Trials’ (SPIRIT), and an additional file is added listing each paragraph and its location in the protocol (Additional file 1). In addition an additional file displays the timing schedule of the enrolment, interventions and assessments (Additional file 2).

### Safety

The study population, which consists of resuscitated OHCA patients admitted to the ICU, is a severely ill group of patients. Most adverse events (AE) are common in this patient group irrespective of treatment strategies. AEs will be recorded daily. Adverse events relevant to the study drug are considered to be death, the need for mechanical hemodynamic support, hypoglycaemia < 3.0 mmol/l, pancreatitis (S-amylase > 3 units per liter), and the need for renal replacement therapy in the first 3 days.

#### Adverse events (AE)

1. Bleeding: From nose, gastrointestinal tract, oral cavity, genitals, insertion sites and intra-muscular and other bleeding
2. Major bleeding: Uncontrolled bleeding (>1 unit of blood/10 kg/hour), bleeding causing fatality, symptomatic bleeding in a critical organ, e.g. intracranial, intraspinal, intraocular, intraarticular or pericardial. In addition, other bleeding, e.g. retroperitoneal, muscular, solid organ or thoracic with haemoglobin < 50 g/l and requiring > 2 units of blood
3. Infection: Severe sepsis, septic shock, pneumonia or other
4. Renal impairment: Need for continuous renal replacement therapy or intermittent haemodialysis
5. Electrolyte disorders: Sustained hyperglycaemia (>10 mmol/l > 4 hours) or hypoglycaemia (<3.0 mmol/l)
6. Arrhythmia: ventricular fibrillation, sustained ventricular tachycardia, tachycardia > 130/min, bradycardia < 40/min, atrial flutter, atrial fibrillation, need for pacing or circulatory collapse mandating cardiopulmonary resuscitation (CPR).
7. Seizures: Tonic-clonic, myoclonic or electrographic status epilepticus.
8. Shivering

Safety variables and AEs will be recorded continuously during the first 7 days and will be reported within 24 hours from awareness of the AE. AEs occurring later than this will be evaluated at the scheduled telephone follow-up calls at 180 days.

#### Serious adverse events (SAE)

For every AE reported in the CRF, an additional question will be asked: Has there been any SAE during the last 24 hours? An SAE is an AE that results in death, is life threatening and requires prolongation of hospitalisation or results in significant disability/incapacity. Uncontrolled bleeding (>1 unit of blood/10 kg/hour), bleeding causing fatality, intracerebral bleeding, septic shock and life threatening arrhythmia mandating CPR will always be considered an SAE.

#### Suspected unexpected serious adverse reactions (SUSAR)

A SUSAR is an unexpected and serious AE with presumed relation to the investigational drug. The term ‘unexpected’ is defined using the Byetta® Summary of product characteristics (http://www.ema.europa.eu/docs/en_GB/document_library/EPAR_-_Product_Information/human/000698/WC500051845.pdf), current as of 19 January 2014, as the reference document. The primary investigator as sponsor is responsible to report all serious (fatal or life threatening) SUSARs to the Danish Health and Medicines Authority as soon as possible and no later than 7 days after having been made aware of such an event. For all other SUSARs, the Danish Health and Medicines Authority must be informed as soon as possible and no later than 15 days after the sponsor has been made aware of the event.

All AEs, SAEs and SUSARs will be recorded and evaluated by the sponsor. The AEs will be reported in the electronic AE form no later than 24 hours from awareness of the AE. Each SAE and SUSAR requires that the investigator fill in the AE form, which is included in the CRFs. The following variables will be recorded: description of the event, onset and end of the event, severity, relation to intervention, action taken and outcome. Any AE occurring during the trial will be treated according to established standards, and the patient will be followed until the event has disappeared or until the condition has stabilised.

### Sample size estimation

The primary outcome is feasibility and the area under the NSE curve (NSE AUC) at 72 hours. Since no difference in feasibility is expected and the trial is designed as a pilot study, we have chosen to power this study according to differences in the NSE AUC. A difference of 20 % is defined as the minimal clinically relevant difference. Previous studies have found a value of approximately 20 μg/ml (25–75 % CI 10–45) at 72 hours [21]. We expect a mean AUC at 72 hours of 50 μg/ml*day, assuming an SD of 30 % of the mean AUC. The α is set at 0.025 (co-primary endpoint), which gives a power of 90 % if 50 patients are included in both groups. A 10 % loss of final measurement is expected (patients expiring before full 72 hours). Therefore, we aim to include 120 patients in total.

### Statistical analysis plan

#### General principles

The general principles for statistical analyses will be:

1. Analyses will be performed according to the intention-to-treat principle with patients lost to follow-up included in the denominator [22].
2. A two-sided significance level of 0.025 will be applied to the primary endpoints and 0.05 will be applied to the secondary endpoints.
3. The two trial sites will be asked to complete all CRFs and other forms if missing data is found in the electronic database. Missing data will be reported in the publication.
4. More than 5 % missing data in outcome variables (see Endpoints) will result in multiple imputation with the creation of 10 imputed datasets to be analysed separately and then aggregated into one estimate of the intervention’s effect on the primary and secondary endpoints [23, 24].

#### Trial profile

A flowchart of the study participants will be displayed according to the Consolidated Standards of Reporting Trials (CONSORT) diagram [25] as Fig. 1.

#### Baseline data

The predefined baseline variables will be as follows:

1. Sex
2. Age
3. Comorbidities (pre-morbid CPC, NYHA class 3 or worse, previous myocardial infarction, ischemic heart disease, previous arrhythmia, previous cardiac arrest, arterial hypertension, transient ischemic attack or stroke, epilepsy, diabetes, asthma or chronic obstructive pulmonary disease, chronic haemodialysis or peritoneal dialysis, hepatic cirrhosis, haematological malignancy, other malignancy, AIDS, alcoholism, intravenous drug abuse, or other immunodeficiency)
4. Previous percutaneous coronary intervention
5. Previous coronary artery bypass graft
6. Previous valvular surgery
7. Implantable cardioverter-defibrillator and/or pacemaker
8. Pre-hospital variables
  - Location of cardiac arrest
  - Bystander witnessed arrest
  - Bystander CPR
  - Shockable primary rhythm
  - Time to basic life support
  - Time to advanced life support
  - Time to ROSC
9. Admission variables
  - First measured temperature
  - Glasgow Coma Score
  - Shock at admission
  - ST-elevation acute myocardial infarction
  - pH
  - Lactate
  - Creatinine

Possible differences in baseline characteristics between the treatment groups will be analysed and displayed in Table 1. Continuous variables will be presented as mean ± SD, and differences will be analysed with the *t* test. In case of skewed data, continuous variables will be presented as median (inter-quartile range) and a *t* test will be applied following logarithmic transformation; secondarily, a non-parametric Mann-Whitney test will be applied. Categorical variables will be presented as *n* (%) and differences will be analysed with the chi-square test.

#### Analysis of endpoints

##### Primary outcome

The primary outcome of feasibility will be reported as the absolute percentage (with 95 % confidence interval) of patients in whom the study drug administration was initiated within 240 minutes of ROSC and the proportion in whom the study drug infusion was completed. The difference in NSE AUC between treatment groups will be tested with the independent-sample *t* test after logarithmic transformation (log2) to approximate a normal distribution. Missing NSE values will be imputed.

##### Secondary and tertiary outcomes

Differences between treatment groups will be analysed with an independent sample *t* test or chi-Square test depending on the variable being numerical or categorical. In case of skewed data, logarithmic transformation will be applied to approximate normal distribution. Crude survival analyses stratified to the treatment groups are performed using proportional hazard models. Hazard rates will also be reported adjusted for site, sex, age, shock-able primary rhythm, and time to ROSC (logarithmically transformed). Hazard ratios will be presented with 95 % confidence intervals.

##### Subgroup analyses

Subgroups will be analysed according to pre-defined design variables: over or under median age, shockable rhythm, sex, the presence of shock at admission, diagnosed AMI and over or under median time from arrest to ROSC. Difference in intervention effect estimates according to subgroup will be declared exclusively based on a statistically significant test of interaction.

### Ethical justification

Participation in the GLP-1 trial will not interfere with or delay routine diagnostic or therapeutic procedures. The trial investigates a potential beneficial effect of the GLP-1 analogue Exenatide, which seems to have effect in various degenerative neurological conditions and has been associated with reduced cerebral infarct size in animal studies. The current knowledge and safety data of intravenous infusion in patients with acute diseases have been referenced above.

The ethical justifications for interventions in the GLP-1 trial are as follows:

1. Knowledge of the neuroprotective effect of GLP-1 analogues in comatose patients resuscitated after OHCA cannot be gained outside the acute setting. Research in a non-acute setting is not possible, and assessment in a human experimental model is obviously unethical.
2. The interventions should be initiated as soon as possible after ROSC to alleviate the reperfusion injury and to reduce progressive brain injury by apoptosis. Therefore, consent from patients is not feasible and awaiting the consent of relatives would induce an unacceptable institutional delay of study drug administration in most cases.
3. The administration of the study drug is considered safe and only exposes the patient to a minimal risk based on previous studies in patients with ST-elevation myocardial infarction [17].
4. Increased knowledge of the therapeutic potential of pharmacological attenuation of anoxic brain damage would increase the scientific knowledge of the condition for the individual and other patients resuscitated from OHCA.
5. Any relevant previously expressed objections to participation in clinical trials by eligible patients known to the researcher will be respected, including the termination of study participation by request from the next of kin.
6. Inclusion in the trial may be of value to the individual patient but is valuable to the group of patients in general because further knowledge is needed to continue the optimisation of neuroprotective interventions in the post-resuscitation phase.

### Publication

The trial’s results will be published in international peer-reviewed journals with no restrictions. Authorship will be granted in accordance with the Vancouver Protocol. The physicians involved in the trial will write the resulting articles, and professional writers will not be used. The study database will be maintained for 15 years. Sebastian Wiberg and Jesper Kjaergaard will be responsible for collecting the data for final analysis. The treatment allocation code will remain concealed until the database is locked. GCP monitoring is performed on the endpoints as well as on the inclusion and exclusion criteria. Analyses and data access will be logged and restricted hereafter. The dataset will be made publically available after 2 years by inquiry to the principal investigator (PI).

## Discussion

Due to the acute setting of this pharmaceutical trial in combination with the serious prognoses for the patients involved, a number of interesting challenges have arisen.

### Adverse events

Primarily, our population consists of severely ill patients with a high mortality rate. This makes adjudication of the AEs, SAEs and SUSARs challenging. Thus, the principal investigator or delegated investigator evaluates and adjudicates AEs on a day-to-day basis and reports to relevant authorities according to Danish legislation. In the event of a suspected SUSAR, the digital algorithm can unblind the treatment allocation of a single patient through the trial website. If this becomes necessary, it will be reported along with the results of the study.

### Surrogate endpoints

Secondly, our primary endpoint defined as area under the NSE-curve at 72 hours is a surrogate marker for poor neurological outcome and death. The surrogate marker is used instead of a hard endpoint in order to power this pilot study adequately. Previously, NSE has been shown to be a solid marker for poor outcome after OHCA in patients undergoing TTM, with an area under the receiver operating characteristics curve of 0.86 [26]. Thus, our surrogate endpoint reflects the clinically relevant question of whether Exenatide has neuroprotective effects in our population.

In conclusion, this article describes the design and planned analysis used in the GLP-1 trial for the first publication of the primary outcomes. This approach minimizes the overall risk of bias and data-driven results.

### Trial status

The trial is currently ongoing.

## Abbreviations

AE, adverse event; AUC, area under the curve; CPC, cerebral performance category; CPR, cardiopulmonary resuscitation; CRF, case report file; GCP, good clinical practice; GLP-1, glucagon-like-peptide 1; ICU, intensive care unit; MI, myocardial infarction; MRI, magnetic resonance imaging; mRS, modified Rankin scale; NSE, neuron-specific enolase; OHCA, out-of-hospital cardiac arrest; ROSC, return of spontaneous circulation; SAE, serious adverse event; SUSAR, suspected unexpected serious adverse reaction; TTM, targeted temperature management

## Additional files

Additional file 1: SPIRIT checklist. Each SPIRIT paragraph has been addressed regarding location in the protocol. (DOC 122 kb) Additional file 2: SPIRIT figure. Schedule of enrolment, interventions, and assessments. (DOC 74 kb)
