# Supplementary material for: GLP-1 analogues for neuroprotection after out-of-hospital cardiac arrest: study protocol for a randomized controlled trial
Source: Trials. 2016 Jun 30;17:304. doi: 10.1186/s13063-016-1421-2 (PMC4929765; doi:10.1186/s13063-016-1421-2)
Supplement: Additional file 2: — SPIRIT figure. Schedule of enrolment, interventions, and assessments. (DOC 74 kb) [file 13063_2016_1421_MOESM2_ESM.doc]

Additional file 1: Figure 1. Schedule of enrolment, interventions and assessments.

|  | **STUDY PERIOD** | | | | | | | | | |
| --- | --- | --- | --- | --- | --- | --- | --- | --- | --- | --- |
|  | **Enrolment** | **Allocation** | **Post-allocation** | | | | | | | **Close-out** |
| **TIMEPOINT**** | ***NLT 240min after ROSC*** | **0** | ***6h15m*** | ***24h*** | ***48h*** | ***72h*** | ***7d*** | ***30d*** | ***90d*** | ***180d*** |
| **ENROLMENT:** |  | | | | | | | | | |
| **Eligibility screen** | X |  |  |  |  |  |  |  |  |  |
| **Informed consent from legal representative** | X |  |  |  |  |  |  |  |  |  |
| **Informed consent from relative and general practitioner** |  | As soon as possible in accordance with Danish Legislation | | | | | |  |  |  |
| **Informed consent from patient** |  |  |  |  | As soon as possible in accordance with Danish Legislation | | | | |  |
| **Allocation** |  | X |  |  |  |  |  |  |  |  |
| **INTERVENTIONS:** |  | | | | | | | | | |
| ***[Exenatide]*** |  |  |  |  |  |  |  |  |  |  |
| ***[Placebo]*** |  |  |  |  |  |  |  |  |  |  |
| **ASSESSMENTS:** |  | | | | | | | | | |
| ***Baseline variables:***  ***Sex, age, comorbidities, PCI, CABG, Valvular surgery, ICD*** | X | X |  |  |  |  |  |  |  |  |
| ***Pre hospital variables:***  ***Location of arrest, witnessed arrest, bystander CPR,*** | X | X |  |  |  |  |  |  |  |  |
| ***Shockable primary rhythm, time to BLS, time to ALS, time to ROSC*** |  |  |  |  |  |  |  |  |  |  |
|  |
| ***Admission variables:***  ***First measured temperature, Glasgow Coma Scale, shock at admission, ST-elevation myocardial infarction, pH, lactate, creatinine*** | X | X |  |  |  |  |  |  |  |  |
| ***Primary end point, feasibility*** |  |  | X |  |  |  |  |  |  |  |
| ***NSE and S100b-measurements*** |  |  |  | X | X | X |  |  |  |  |
| ***Vital status*** |  |  |  |  |  | X | X | X | X | X |
| ***Cerebral Performance Category*** |  |  |  |  |  |  | X | X | X | X |
| ***modified Rankin Scale*** |  |  |  |  |  |  | X | X | X | X |
| ***Serious Adverse Events*** |  |  |  |  |  |  |  |  |  |  |
| ***Troponin T and CKMB*** |  | X |  | X |  |  |  |  |  |  |
| ***Best LVEF*** |  |  |  |  |  |  |  |  |  |  |
